# Pigs lacking *TMPRSS2* displayed fewer lung lesions and reduced inflammatory response when infected with influenza A virus

**DOI:** 10.3389/fgeed.2023.1320180

**Published:** 2024-05-31

**Authors:** Giovana Ciacci Zanella, Celeste A. Snyder, Bailey L. Arruda, Kristin Whitworth, Erin Green, Ravikanth Reddy Poonooru, Bhanu P. Telugu, Amy L. Baker

**Affiliations:** ^1^ Virus and Prion Research Unit, National Animal Disease Center, United States Department of Agriculture, Agricultural Research Service, Ames, IA, United States; ^2^ Department of Veterinary Microbiology and Preventive Medicine, College of Veterinary Medicine, Iowa State University, Ames, IA, United States; ^3^ National Swine Resource and Research Center, University of Missouri, Columbia, MO, United States; ^4^ Division of Animal Sciences, College of Agriculture, Food and Natural Resources, University of Missouri, Columbia, MO, United States

**Keywords:** influenza A, swine, *TMPRSS2* gene, knockout, somatic cell nuclear transfer, proinflammatory response, RNAscope

## Abstract

Influenza A virus (IAV) infection is initiated by hemagglutinin (HA), a glycoprotein exposed on the virion’s lipid envelope that undergoes cleavage by host cell proteases to ensure membrane fusion, entry into the host cells, and completion of the viral cycle. Transmembrane protease serine S1 member 2 (TMPRSS2) is a host transmembrane protease expressed throughout the porcine airway epithelium and is purported to play a major role in the HA cleavage process, thereby influencing viral pathogenicity and tissue tropism. Pigs are natural hosts of IAV and IAV disease causes substantial economic impact on the pork industry worldwide. Previous studies in mice demonstrated that knocking out expression of *TMPRSS2* gene was safe and inhibited the spread of IAV after experimental challenge. Therefore, we hypothesized that knockout of *TMPRSS2* will prevent IAV infectivity in the swine model. We investigated this hypothesis by comparing pathogenesis of an H1N1pdm09 virus challenge in wildtype (WT) control and in *TMPRSS2* knockout (*TMPRSS2* ^−/−^) pigs. We demonstrated that *TMPRSS2* was expressed in the respiratory tract in WT pigs with and without IAV infection. No differences in nasal viral shedding and lung lavage viral titers were observed between WT and *TMPRSS2* ^−/−^ pigs. However, the *TMPRSS2* ^−/−^ pig group had significantly less lung lesions and significant reductions in antiviral and proinflammatory cytokines in the lung. The virus titer results in our direct challenge model contradict prior studies in the murine animal model, but the reduced lung lesions and cytokine profile suggest a possible role for TMPRSS2 in the proinflammatory antiviral response. Further research is warranted to investigate the role of TMPRSS2 in swine IAV infection and disease.

## Introduction

Influenza A virus (IAV) is responsible for one of the most important viral respiratory diseases in pigs and humans (Anderson et al., 2021). Pigs are natural hosts of IAVs and while the clinical signs of uncomplicated infection may be mild, swine influenza is responsible for substantial economic impact on the pork industry worldwide due to multi-etiology porcine respiratory disease complex and predisposition of pigs to secondary respiratory infections (Gorres et al., 2011). Pigs possess cell-surface receptors preferred by both avian and human IAV and are susceptible to infection by certain human and avian IAV strains (Matrosovich et al., 2009). Therefore, they are considered “mixing vessels” for the potential to host co-infection with endemic swine adapted strains and human seasonal or avian strains, which may lead to reassortment and generation of potentially novel IAV strains. The 2009 influenza pandemic was a reminder of this possibility, when a novel swine-origin H1N1 (pdm09) IAV that contained ancestral gene segments from human, avian and swine IAV emerged and rapidly spread within the human population worldwide, reaching pandemic status (Henningson et al., 2015). The pdm09 became a seasonal strain in humans and continues to transmit from humans to pigs, greatly increasing the reassortment and evolution potential of swine IAV (Markin et al., 2023). Therefore, control of IAV in swine will have a downstream impact on human public health.

Infection of the host by IAV is initiated by binding of hemagglutinin (HA), a glycoprotein exposed on the IAV lipid envelope to the cognate cell surface receptor and fusing with cellular endosomal membrane. However, the HA is synthesized as a precursor protein HA0 necessary for trimerization and binding to host receptors but requires proteolytic cleavage by host cellular proteases into HA1 (peripheral N-terminal) and HA2 (C-terminal transmembrane) fragments to acquire competence for fusion following cell entry. The proteolytic cleavage of HA results in a cascade of events, first rendering the HA fragment metastable, inducing major conformational changes in low endosomal pH, exposing the fusion peptide, and ultimately fusing with the endosomal membrane to release IAV ribonucleoproteins (RNP) into the cytoplasm (Peitsch et al., 2014; Wang et al., 2022). The cleavage of the HA by host cell proteases is the rate limiting step for viral infectivity and determining pathogenicity and tissue tropism (Bottcher et al., 2006; Hatesuer et al., 2013). *In vitro* and *in vivo* studies demonstrated that type II transmembrane proteases are responsible for cleavage at the monobasic site of HA and IAV infection in human, mice, and swine airway epithelium (Peitsch et al., 2014; Wang et al., 2022). TMPRSS2 is a type II transmembrane serine protease and is indicated to be one of the serine proteases responsible for HA0 proteolytic cleavage of several IAV HA subtypes that contain an arginine in the linker peptide between HA1 and HA2 domains, as shown for H1N1 (A/PR/8/34) and H7N9 (A/Anhui/1/13) (Sakai et al., 2014; Tarnow et al., 2014). In mice, knockout of *TMPRSS2* resulted in the protection against H1N1 (A/PR/8/34), mouse adapted H1N1 (A/California/04/09) and H7N9 (A/Anhui/1/13) IAV infections (Sakai et al., 2014; Tarnow et al., 2014), and did not result in overt morbidity or mortality (Kim et al., 2006). Likewise, in humans, genetic linkage studies have identified genetic variants in patients that have high *TMPRSS2* expression exhibited a higher risk for severe H1N1 (pdm09) and Asian H7N9 IAV infection (Cheng et al., 2015). These results highlight the growing evidence supporting the role of TMPRSS2 in IAV infectivity.

Pigs express TMPRSS2 and other HA cleaving proteases in different parts of the respiratory tract, including the epiglottis, trachea, and lung (Peitsch et al., 2014). Since IAVs are a threat to animal and public health, measures to control on-going circulation and viral evolution in the swine population are essential. Disease resistant animal models, including pigs, have been successfully generated over recent years through genetic modifications (Whitworth et al., 2022). Our working hypothesis was that TMPRSS2 plays an important role in IAV infection, and knockout of the *TMPRSS2* gene regulates IAV infectivity in the swine model. We investigated this hypothesis by generating *TMPRSS2* knockout (*TMPRSS2* ^*−/−*^) gene edited pigs and comparing the pathogenesis of an H1N1pdm09 strain in wildtype (WT) control and *TMPRSS2* ^*−/−*^ pigs.

## Materials and methods

### Expression of *TMPRSS2* in wildtype swine respiratory tract

To demonstrate the cellular distribution of *TMPRSS2* mRNA expression in normal swine respiratory tissues, lung and trachea tissue sections from 4 non-infected and 4 IAV infected WT pigs euthanized on 5 days post inoculation (dpi) were cut, fixed in fresh 10% neutral-buffered formalin for 48 h, transferred to 70% ethanol, embedded in paraffin at room temperature, and mounted on Superfrost^®^ Plus slides (Avantor, Radnor, PA). RNAscope^®^ assay was performed according to the manufacturer’s protocol for the automated Ventana Discovery™ Ultra system (Advanced Cell Diagnostics, Newark, CA). Antigen retrieval was carried out for 24 min at 97°C followed by Protease Plus pre-treatment for 16 min at 37°C, and incubation with the amp reagent for 24 min. The target probe was RNAscope™ 2.5 VS *Sus scrofa* transmembrane protease serine 2 mRNA (Ss-TMPRSS2-C2). The positive control probe was RNAscope™ 2.5 VS *Sus scrofa* peptidylprolyl isomerase B cyclophilin B (Ss-PPIB) partial mRNA and the negative control probe was RNAscope™ 2.5 VS *Bacillus subtilis* strain SMY methylglyoxal synthase (mgsA) gene, partial coding sequence. Slides were then counterstained, cover slipped, and stored in the dark until image evaluation.

### Generation of *TMPRSS2* ^−/−^ fibroblasts

Somatic cells null for *TMPRSS2* were derived from genome edited pigs reported in Whitworth et al. (Whitworth et al., 2017). Briefly, a combination of CRISPR/Cas9 *in vitro* transcribed RNA and two single guide RNAs (CRISPR guides #3 and #6) flanking an essential exon (exon #2) that contains the “ATG” start codon and coding sequence, were co-injected into cytoplasm of presumptive zygotes 14 h post fertilization. Following embryo transfer and birth of edited offspring, the founder boar was bred with WT animal, and the line was propagated by breeding. Following subsequent cross of heterozygous animals, fibroblasts null for *TMPRSS2* were obtained from ear and tail skin biopsies of *TMPRSS2* ^*−/−*^ piglets. The tissue samples were cut into small pieces (of ∼1 mm^3^) in 100 mm cell culture dish and digested in 20 mL of the digestion medium (DMEM HG 15% fetal bovine serum [FBS] + Collagenase [200 μg/mL type IV, sigma C5138] + Dnase I [25 Kunitz/mL, sigma D-4263] + antibiotics [1 μg/mL gentamicin, 10 mg/mL stock]) for 7–8 h in a tissue culture incubator at 38.5°C in 5% CO_2_ and air. The fibroblast cultures were then cryopreserved in 10% DMSO in DMEM and stored in liquid nitrogen until pigs were recreated by somatic cell nuclear transfer (SCNT). One male and 1 female line (94–2 and 94–3, respectively) were used for SCNT. The edit for the 94–2 line included a 211 bp deletion and a 11 bp insertion in exon 2 removing the start codon on one allele and an 875 bp deletion removing the entire exon 2 on the other allele. The edit for the 94–3 line included a 210 bp deletion and a 17 bp insertion in exon 2, removing the start codon on one allele and an 875 bp deletion removing the entire exon 2 on the other allele.

### Generation of *TMPRSS2* ^*−/−*^ pigs *via* somatic cell nuclear transfer (SCNT)

*TMPRSS2* ^*−/−*^ pigs were generated by SCNT as described in our previous studies (Park et al., 2021). Briefly, cumulus-oocyte-complexes were purchased from a commercial supplier (De Soto Biosciences, Seymour, TN, United States). After *in vitro* maturation, the cumulus cells were removed from oocytes by gentle pipetting in a 0.1% (w/v) hyaluronidase solution. The oocytes were enucleated by aspirating the polar body and the MII metaphase plates by a micropipette (Humagen, Charlottesville, VA, United States) in 0.1% DPBS supplemented with 5 μg/mL of cytochalasin B. After enucleation, a donor cell was placed into the perivitelline space of an enucleated oocyte. The cell–oocyte couplets were fused by applying two direct current (DC) pulses (1-s interval) of 2.0 kV/cm for 30 μs using an ECM 2001 Electroporation System (BTX, Holliston, MA). After fusion, the reconstructed oocytes were activated by a DC pulse of 1.0 kV/cm for 60 µs, followed by post-activation in 2 mM 6-dimethylaminopurine for 3 h. After overnight culture in porcine zygote medium with 0.3% bovine serum albumin (BSA) (PZM3) with a histone deacetylase inhibitor Scriptaid (0.5 μM), the cloned embryos were cultured and maintained in PZM3 medium in a low oxygen environment (5% O_2_, 5% CO_2_ and 90% N_2_) (Redel et al., 2019). Embryos that progressed to blastocysts were surgically transferred into synchronized recipients on the days 3–5 following standing estrus. Seven embryo transfers resulted in 2 pregnancies and 7 live piglets from 2 litters. Two piglets were male and derived from the 94–2 line and 5 piglets were female and derived from 94–3 line. Piglets were genotyped to confirm *TMPRRS2* ^*−/−*^ status by PCR as described previously (Whitworth et al., 2017).

### Virus

The IAV strain used in the animal study was A/swine/Iowa/A02524480/2020 (IA/20), a representative of the 1A.3.3.2 H1N1pdm09 lineage of IAV. This H1N1 phylogenetic clade pairing represented approximately 5% of the IAV detected in swine in the U.S. in 2020–2023, and the remaining 6 gene segments represent one of the most common whole genome constellation patterns in US swine in the last 3 years (TTTPPT) (Arendsee et al., 2021). The HA and neuraminidase (NA) genes originated from a human seasonal H1N1 of the pdm09 lineage circulating in the 2018–2019 influenza season that spilled into pigs and sustained transmission in the animal population. The IA/20 isolate originated from a clinical case of respiratory disease in pigs and was obtained from the IAV repository at the National Veterinary Services Laboratories (NVSL) through the U.S. Department of Agriculture (USDA) IAV swine surveillance system in conjunction with the USDA-National Animal Health Laboratory Network (NAHLN). Prior to challenge, the virus was propagated and titrated in Madin-Darby Canine Kidney (MDCK) cells.

### *In vivo* animal study

Control WT pigs were obtained from a commercial source free of porcine reproductive and respiratory syndrome virus, *Mycoplasma* hyopneumoniae and IAV, and used as positive and negative controls for IAV infection. The *TMPRSS2* ^−/−^ pigs were obtained from the National Swine Resource and Research Center (RRID: NSRRC:0060), from two separate litters with *N* = 2 and *N* = 5. Pigs were implanted with a Life Chip from Bio-Thermo Technology (Destron Fearing, DFW Airport, Texas), separated into three groups in separate containment rooms, one group of negative WT control pigs (non-challenged) (*N* = 5), a group of positive control WT pigs (*N* = 10), and the *TMPRSS2* ^*−/−*^ group (*N* = 7). After arrival, all the pigs were housed in bio-safety level 2 containment and cared for in compliance with the Institutional Animal Care and Use Committee of the National Animal Disease Center. The pigs were treated with ceftiofur crystalline free acid and tulathromycin (Zoetis Animal Health, Florham Park, NJ) to reduce secondary bacterial infections. All were screened by a commercial ELISA kit to ensure absence of IAV antibody (Swine Influenza Virus Antibody Test, IDEXX, Westbrook, ME). Temperature collection commenced 2 days before challenge and continued until 5 days post inoculation (dpi). At 4–5 weeks of age, pigs were inoculated intratracheally and intranasally with 2 mL and 1 mL, respectively, of 1 ×10^5^ 50% tissue culture infectious dose (TCID_50_)/mL of the IA/20 virus. Inoculation was done under sedation using an intramuscular injection of ketamine (8 mg/kg; Phoenix, St. Joseph, MO), xylazine (4 mg/kg; Lloyd Inc., Shenandoah, IA), and Telazol (6 mg/kg; Zoetis Animal Health, Florham Park, NJ) cocktail. Nasal swabs (NS) were collected daily from 0 to 5 dpi, in infection media containing 2 mL minimum-essential-medium (MEM) supplemented with 1:1000 TPCK Trypsin, to evaluate nasal viral shedding.

On 5 dpi the pigs were bled and humanely euthanized with a lethal dose of pentobarbital (Fatal Plus; Vortech Pharmaceuticals, Dearborn, MI). Lungs were aseptically removed, evaluated for macroscopic lesions and lavaged with 50 mL of MEM containing 1% BSA to obtain bronchoalveolar lavage fluids (BALF). Right cardiac or affected lung lobe and distal trachea tissues were collected from all pigs.

### Confirmation of *TMPRSS2* ^*−/−*^ knockout

Lung tissues collected during necropsy were maintained in RNA*later®* (R0901, Sigma-Aldrich). Isolation of genomic DNA (gDNA) from tissues was done with Monarch^®^ Genomic DNA purification kit (New England Biolabs, Cat. No. T3010L) for DNA isolation as per manufacturer’s recommended protocol. All Prep DNA/RNA/Protein Mini Kit (Qiagen, Cat. No./ID: 80004) for mRNA isolation as per the recommended protocol. Synthesis of cDNA was performed using an Applied BiosystemsTM High-Capacity RNA-to-cDNATM kit (ThermoFisher, Cat. No. 4387406) for cDNA synthesis as per the manufacturers’ recommended protocol. Genotyping PCR was performed using TaqPolymerase mix (Bioline) by using the following conditions: denaturation and polymerase activation step of 95°C for 1 min, 35 cycles of 94°C for 30 s (denaturation), 52°C for 30 s (annealing), 72°C for 1 min (extension), and the final extension step of 72°C for 5 min. RT-PCR was performed by using KOD Hot start mastermix (Novagen) by using the following conditions. For ACTG1: denaturation and polymerase activation step of 95°C for 2 min, 35 cycles of 95°C for 20 s (denaturation), 60°C for 15 s (annealing), 72°C for 2 min (extension), and the final extension step of 72°C for 2 min. The primer sequence and information are provided in Supplementary Table S1.

### Virologic assays and PCR antigen detection

All nasal swab samples were filtered with a 0.2-micron syringe filter and plated onto 48-well plates containing confluent MDCK cells that were washed twice with phosphate-buffered saline (PBS) and cultured at 37°C for 48–72 h, depending on the amount of cytopathic effect present. Plates were fixed and stained for immunocytochemistry (ICC) as previously described (Gauger and Vincent, 2014). BALF samples were streaked (100 µL) on Casmin (NAD-enriched) and blood agar plates for 48 h at 37°C to verify lack of significant aerobic bacterial growth. Virus isolation-positive nasal swabs and BALF samples were analyzed for viral titers by being plated onto 96-well plates of MDCK cells in 10-fold serial triplicate dilutions in 100 µL with infection media containing antibiotics. At 48 h, plates were fixed and stained (Gauger and Vincent, 2014). Titers were calculated for each sample as TCID_50_ per mL and transformed to log_10_ for comparison purposes. Nasal swab samples were subjected to RNA isolation using MagMAX-96 Viral RNA Isolation Kit and qPCR with VetMAXTM-Gold SIV Detection Kit (Thermo Fisher Scientific, Waltham, MA) to compare cycle threshold (Ct) values.

### Pathologic changes and immunohistochemical staining for IAV

At necropsy, the percentages of lung surface with lesions typical of IAV infection were recorded. A visual estimation was made for each lung lobe, and a total percentage for the entire lung was calculated based on weighted proportions of each lobe to the total lung volume (Halbur et al., 1995). All tissues collected and fixed in 10% formalin were transferred to 70% ethanol buffer approximately 48 h following collection. Lung and tracheal tissues were processed by routine histologic procedures and the slides were stained with hematoxylin and eosin. Microscopic lesions were scored by a veterinary pathologist with parameters previously described (Morgan et al., 2016). Immunohistochemical (IHC) staining was carried out manually on 5-µm thick lung and tracheal sections, using a primary antibody that targets the IAV nucleoprotein (NP). Heat-induced epitope retrieval was performed in an EZ-retriever^®^ IR System (BioGenex, Fremont, CA) using a citrate buffer (pH 6; Abcam, Boston, MA) heated to 95°C for 2 cycles of 10 min. Slides were allowed to cool for 20 min followed by a rinse with deionized water and Dako Wash Buffer (2 × 5 min: Agilent, Santa Clara, CA). Slides were then incubated with hydrogen peroxide (3% in PBS; 2 × 8 min) to quench endogenous peroxidase activity, washed, and blocked for 60 min with 10% goat serum diluted in Dako Wash Buffer. Slides were then incubated at room temperature for 60 min with primary antibody (1:4000; GTX125989, GeneTex, Irvine, CA) diluted in antibody dilution buffer (S080983-2, Agilent, Santa Clara, CA), washed, and incubated with a secondary antibody (EnVision + anti-rabbit Poly HRP-IgG, Agilent, Santa Clara, CA) for 30 min. Diaminobenzidine chromogen detection was completed using Dako DAB Plus (5 min, Agilent, Santa Clara, CA) and Dako DAB Enhancer (3 min, Agilent, Santa Clara, CA). Slides were then rinsed with deionized water, cleared through gradient alcohol and xylene, counterstained using hematoxylin and cover slipped. Sections were scored by a veterinary pathologist blinded to the study using a 0–4 scoring factor (Gauger et al., 2012) with modifications based upon the quantity of antigen-positive cells present in the tracheal epithelium and either in the conducting airways of the lung, alveolar lumen and/or septa (0 = none, 1 = minimal, 2 = mild, 3 = moderate and 4 = severe or abundant).

### Cytokine and chemokine analysis

BALF samples were centrifuged to remove cellular debris and tested with the Invitrogen ProcartaPlex™ Porcine Cytokine and Chemokine 1 9-Plex (Thermo Fisher Scientific, Waltham, MA) per manufacturer’s instructions. The media used to collect the lavages was used to prepare the standard mix. All samples were assessed on a Luminex MAGPIX Instrument System (Thermo Fisher Scientific, Waltham, MA) for the expression of IFN-α, IFN-γ, IL-1β, IL-10, IL-12/IL-23p40, IL-4, IL-6, IL-8 and TNF-α. The average of duplicate wells for each sample was used for statistical analysis.

### Statistical analysis

Macroscopic pneumonia scores, microscopic tracheal and lung lesion scores, ICC staining scores, log_10_ transformed BALF, nasal swab virus titers and cytokine data were analyzed using ordinary one-way analysis of variance (ANOVA) with Tukey’s multiple comparisons test and IHC scores were analyzed by *t*-test (GraphPad Prism, GraphPad Software, La Jolla, CA). Comparisons were made between groups at the same time-point using a 5% level of significance (*p*-value <0.05) to assess statistical differences. Clinical data associated with this study are available for download from the USDA Ag Data Commons at https://doi.org/10.15482/USDA.ADC/1529793.

## Results

### Confirmation of *TMPRSS2* knockout

The *TMPRSS2* ^*−/−*^ piglets generated by SCNT were genotyped to confirm the expected loss of exon 2 and loss of expression by RT-PCR, respectively using gene specific primers (Supplementary Table S1; Supplementary Figure S1). As shown in Supplementary Figure S2, loss of exon 2 results in the loss of the canonical start codon and truncated polypeptide sequence to render the TMPRRS2 protein non-functional.

### *TMPRSS2* expression in swine respiratory tissue

**FIGURE 1:**
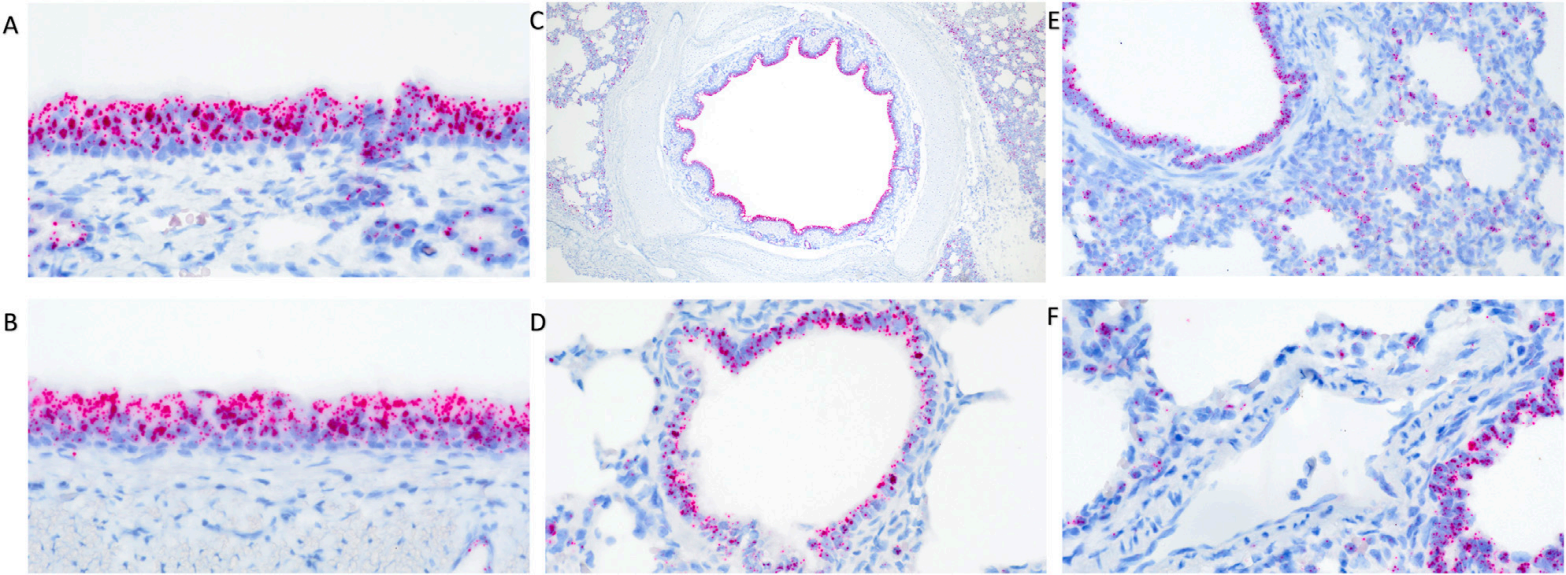
RNAscope detection of *TMPRSS2* transcripts in the respiratory tract of clinically healthy wildtype pigs without IAV infection. There was abundant detection in pseudostratified ciliated epithelium of the turbinate **(A)** and trachea **(B)** and circumferential detection within the respiratory epithelium lining the mainstem bronchi **(C)** and bronchioles **(D)**. Transcripts were detected in cells comprising the alveolar septa **(E)** but rarely detected in endothelial cells **(F)**. Original magnifications: ×600 **(A,B,F)**; 40× **(C)**; 400× **(D,E)**.

In clinically healthy WT pigs, abundant *TMPRSS2* transcripts were detected in the pseudostratified ciliated epithelium of the turbinate (Figure 1A) and trachea (Figure 1B). Similarly, abundant transcripts were circumferentially detected within the respiratory epithelium lining the mainstem bronchi (Figure 1C), bronchioles (Figure 1D), and terminal bronchioles. Transcripts were also commonly detected in cells comprising or within the alveolar septa (Figure 1E) and rarely detected in endothelial cells (Figure 1F). The epithelium of submucosal glands in the turbinate, trachea, and lung also contained transcripts. Multiple transcripts per cell were commonly present with minimal to mild variation between cells in all tissues evaluated.

**FIGURE 2:**
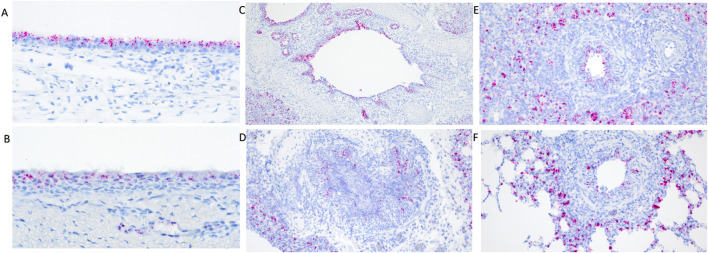
RNAscope detection of *TMPRSS2* transcripts in the respiratory tract of IAV infected wildtype pigs at 5 days post inoculation (dpi). Diminished transcripts were observed in areas of erosion in the turbinate **(A)** and trachea **(B)**. Differential visual observation of TMPRSS2 transcripts was noted in bronchi **(C,D)** and bronchioles **(E,F)** affected by necrotizing bronchiolitis and bronchitis after IAV infection compared to non-challenged pigs. Increased transcription was observed in cells surrounding affected bronchioles **(E,F)** when compared to healthy tissue. Original magnifications: ×200 **(D–F)**; 100× **(C)**; 400× **(A,B)**.

Differential transcript distribution was visually observed in IAV infected WT pigs compared to non-challenged pigs. Diminished expression signal was visually detected in areas of erosion in the turbinate (Figure 2A) and trachea (Figure 2B). Bronchi and bronchioles affected by necrotizing bronchitis (Figures 2C,D) and bronchiolitis (Figures 2E,F) had diminished *TMPRSS2* transcripts with fewer cells containing *TMPRSS2* transcripts. Increased transcription was observed in cells surrounding affected bronchioles (Figure 2F). Differential endothelial transcription was not observed. It is not clear if this differential distribution was a result of downregulation in the expression of *TMPRSS2* or a direct consequence of epithelial cell death due to viral infection. Tissue samples from the *TMPRSS2* ^*−/−*^ were not analyzed by RNAscope assay as the RNA expression is not disrupted, and the RNAscope probes did not target the deleted region.

### Absence of TMPRSS2 had minimal impact on nasal viral shedding

**FIGURE 3:**
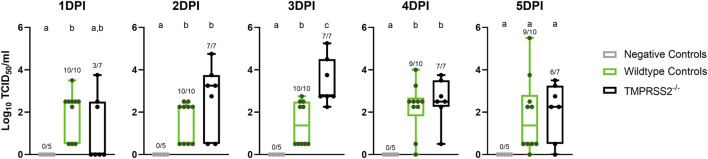
Nasal shedding of A/swine/Iowa/A02524480/2020 (IA/20) H1N1 by virus titration on cell culture in wildtype (WT) and *TMPRSS2* ^*−/−*^ pigs. Virus titers showed as log^10^ transformed group mean TCID50/ml ± standard error of the mean from nasal swabs of 1–5 days post infection in negative controls (grey, *N* = 5), wildtype (green, *N* = 10), or *TMPRSS2* ^*−/−*^ pigs (black, *N* = 7). Point of detection was 0.5. Numbers above the error bars show the number of positive pigs/total pig numbers in group and different lower-case letters (a, b, c) indicate statistical difference between group means (*p* ≤ 0.05) by ordinary one-way analysis of variance (ANOVA) with Tukey’s multiple comparisons (GraphPad Prism, GraphPad Software, La Jolla, CA).

No IAV was detected in nasal secretions from any inoculated pigs at 0 dpi, and all negative control pigs remained negative throughout the study. Both challenged groups had onset of nasal shedding at 1 dpi with 10 of 10 positive in the WT control group, and 2 of 7 in the *TMPRSS2* ^−/−^ group positive for virus on 1 dpi (Figure 3). All 7 pigs in the *TMPRSS2* ^*−/−*^ group were shedding on 2 dpi. Viral shedding detected in nasal swabs continued in both groups throughout the duration of the study. The magnitude of virus titers were significantly different between groups on 3 dpi, when the *TMPRSS2* ^−/−^ pig group had higher mean titers in nasal swabs compared to the WT positive control group (*p* < 0.005). Viral RNA levels were also higher for the *TMPRSS2* ^−/−^ pigs on dpi 2, reflected in significantly lower Ct values (*p* < 0.05).

### Absence of TMPRSS2 resulted in fewer lung lesions despite equivalent virus titers and clinical signs

**FIGURE 4:**
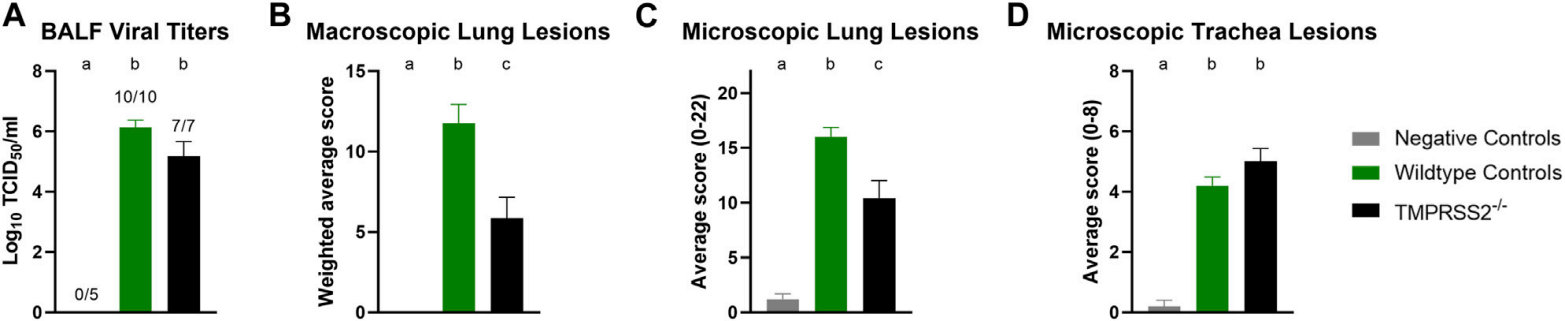
Infection of IA/20 H1N1 in WT and *TMPRSS2* ^*−/−*^ pigs. **(A)** Bronchioalveolar lavage fluid (BALF) TCID_50_/ml viral titers log^10^ transformed. **(B)** Weighted percentage of macroscopic lung lesion. **(C)** Group average microscopic lung scores. **(D)** Group average microscopic trachea scores. Negative controls (grey, *N* = 5), wildtype (green, *N* = 10) and in *TMPRSS2* ^*−/−*^ pigs (black, *N* = 7). Different lower-case letters (a, b, c) indicate statistical difference between group means (*p* ≤ 0.05) by ANOVA with Tukey’s multiple comparisons (GraphPad Prism, GraphPad Software, La Jolla, CA).

**FIGURE 5:**
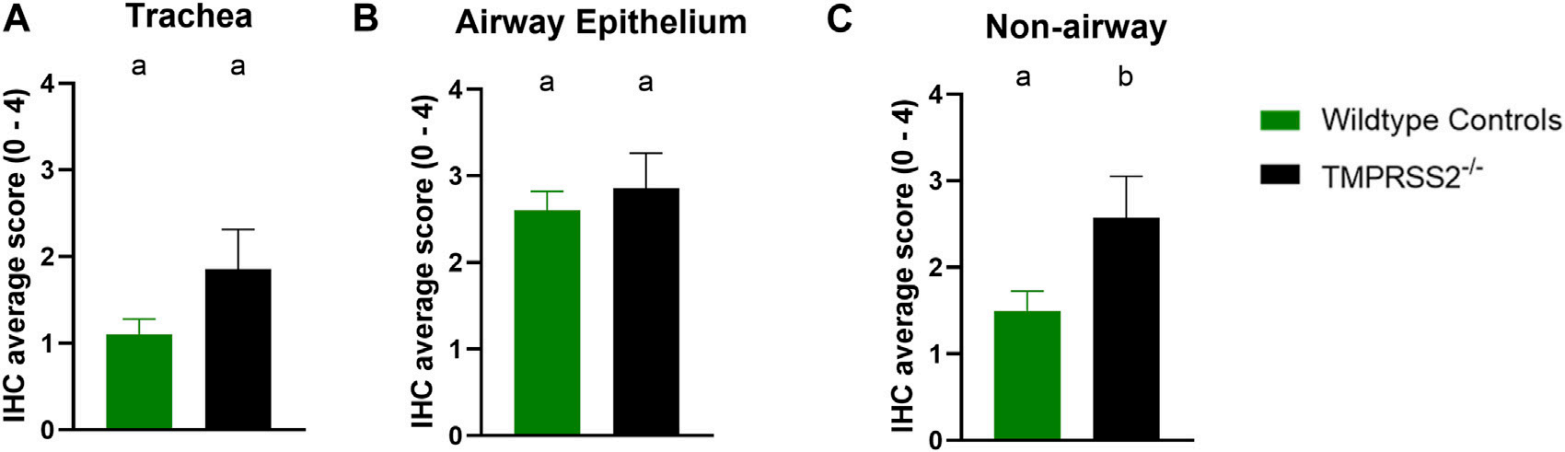
Immunohistochemical (IHC) staining of Influenza A virus nucleoprotein in wildtype (green, N = 10) and *TMPRSS2* ^*−/−*^ (black, N = 7) pig respiratory tract tissue. **(A)** IHC group average score with standard error of the mean from tracheal epithelium. **(B)** Group average with standard error of the mean from conducting airway epithelium of the lung. **(C)** Group average with standard error of the mean from non-airway cells including pneumocytes, macrophages and exudate of the lung. Different lower-case letters (a, b) indicate statistical difference between group means (*p* ≤ 0.05) by *t*-test (GraphPad Prism, GraphPad Software, La Jolla, CA).

Daily average body temperatures were not significantly different between WT and *TMPRSS2* ^*−/−*^ groups at any collection point (Supplementary Table S2). BALF were collected and titrated to evaluate viral replication in the trachea and lungs. All challenged pigs had IAV detected in their BALF samples and mean viral titers were not significantly different between groups (Figure 4A). The IA/20 H1N1 isolate induced mild to moderate percentages of macroscopic pneumonia in all challenged pigs with varying degrees of multifocal to coalescing areas of consolidation, consistent with experimental infection of IAV. Macroscopic lung lesions demonstrated higher average percentages in the WT group compared to the *TMPRSS2* ^*−/−*^ group (Figure 4B; Supplementary Figure S3). Overall, microscopic lung lesions scores followed the same pattern, with the mean histologic lesion scores being significantly lower in the *TMPRSS2* ^*−/−*^ group (Figure 4C). However, tracheal lesions were not significantly different between groups (Figure 4D). IHC staining of tissue sections revealed antigen-positive cells in the lung, trachea, and nasal turbinate tissue of all *TMPRSS2* ^*−/−*^ pigs. When compared, no significant difference in the IHC score was found in the trachea (Figure 5A) or conducting airway epithelium of the lung (Figure 5B) between the *TMPRSS2* ^*−/−*^ and WT pigs. A significant difference between the non-airway IAV antigen-positive cell scoring of the *TMPRSS2* ^*−/−*^ and WT lungs was observed (Figure 5C). The *TMPRSS2* ^*−/−*^ group had higher levels of signal in pneumocytes, macrophages and alveolar luminal exudate than the WT group of pigs. These results indicate that the absence of *TMPRSS2* expression does not impede IAV infection of the upper and lower respiratory tract.

### *TMPRSS2* ^−/−^ pigs had decreased proinflammatory response

**FIGURE 6:**
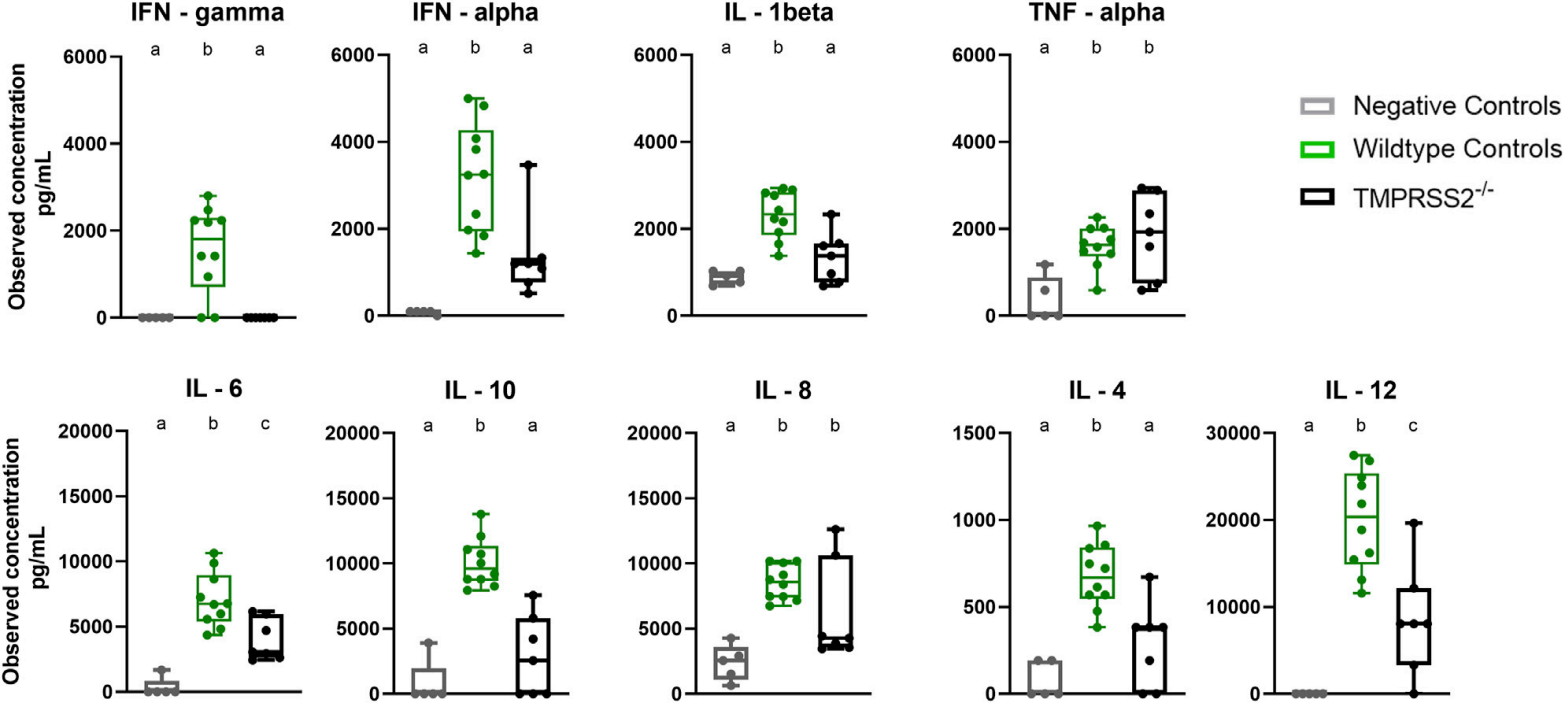
Cytokine and chemokine panel performed on bronchioalveolar lavage fluid (BALF) collected from negative controls (grey, N = 5), wildtype (green, N = 10) and *TMPRSS2* ^*−/−*^ (black, N = 7) pigs 5 days post infection with IA/20 H1N1 virus. Different lower-case letters (a, b, c) indicate statistical difference between group means (*p* ≤ 0.05) by ANOVA with Tukey’s multiple comparisons (GraphPad Prism, GraphPad Software, La Jolla, CA).

BALF samples collected during necropsy at 5 dpi were assessed for cytokine and chemokine proteins with a multiplex bead-based assay. The *TMPRSS2* ^*−/−*^ pigs had elevated levels of IL-6, IL-8, IL-12, and TNF-α compared to the non-challenged negative control group. However, the WT positive challenged control group had significantly higher levels of IFN-α, IFN-γ, IL-1β, IL-6, IL-12, IL-10, and IL-4 when compared to the *TMPRSS2* ^*−/−*^ group (Figure 6). IL-8 quantity was also higher in the WT group, but not statistically significant due to the variability between *TMPRSS2* ^*−/−*^ animals. TNF-α levels were not significantly different between challenged groups.

## Discussion

IAV are known to infect a wide range of animal species, including humans, birds, and pigs (Anderson et al., 2021). Among these, the swine population plays a crucial role in the ecology of IAV, because pigs can be infected by both avian and human IAV. Pigs also act as mixing vessels, allowing for the reassortment and consequently emergence of new viruses. These reassorted novel viruses can carry gene segments from different viral lineages that were previously found in separate animal species. As a result, if returned to their original host, they have the potential to evade existing population immunity and cause disease. Reducing the burden of IAV infection in swine improves the overall welfare of animals by decreasing clinical respiratory disease. This, in turn, protects the economic stability of the swine industry since IAV outbreaks have a significant economic impact due to reduced growth rates, decreased productivity, and increased mortality among infected animals. Reducing the circulation of IAV in pigs also decreases the likelihood of viral reassortment and evolution in swine and consequently the risk of human exposure. The typical approach to reduce IAV burden in the swine population is to implement control measures such as biosecurity and vaccination practices (Vincent et al., 2014). However, due to the complex ecology of swine IAV and the continued evolution and co-circulation of distinct lineages evading control measures, alternative approaches to control influenza circulation require consideration.

Disease-resistant or tolerant animal models exhibit a natural or engineered decrease in susceptibility to specific diseases. They are valuable models to study disease mechanisms, therapeutic interventions, and vaccines (Whitworth et al., 2022). Recently, viral disease resistant pig models have been successfully generated through gene editing technology (Whitworth et al., 2016) and they provide an unprecedented opportunity for deciphering infection dynamics and host-pathogen interactions. Gene edited pigs provide opportunities to mitigate economic losses and maintain a healthier and more productive swine herd and thereby more sustainable farming practices. If pigs are less susceptible to influenza, the risk of transmission to humans will decrease, potentially preventing future outbreaks and pandemics. The swine model is also valuable to study multifaceted host-pathogen interactions that occur during IAV infection because swine are a natural host and share respiratory and physiological similarities with humans (Rajao and Vincent, 2015). Our *TMPRSS2* ^*−/−*^ pig model was created to address the role of TMPRSS2 in swine influenza pathogenesis but may also improve our knowledge of therapeutic approaches to human influenza disease.

The extent of the physiological roles of TMPRSS2 is currently unknown (Antalis et al., 2011); however, it is noteworthy that the lack of TMPRSS2 was not associated with severe unwanted side effects and *TMPRSS2* ^*−/−*^ pigs, similar to the knockout mice, did not exhibit any overt health abnormalities and did not show any developmental alterations such as splayed legs, macroglossia, or low birth weight (Supplementary Figure S4) (Hatesuer et al., 2013; Sakai et al., 2014). In this study, we showed subtle effects of loss of *TMPRSS2* compared to similar studies performed previously in the mouse model (Hatesuer et al., 2013; Sakai et al., 2014). In our swine model, the host-protease TMPRSS2 was not solely responsible for susceptibility to IAV infection. The H1N1 IAV strain tested here was capable of infecting and causing disease in pigs despite the gene editing to prevent *TMPRSS2* expression. The similarity in viral titers from the WT and *TMPRSS2* ^*−/−*^ nasal swabs and BALF samples indicate that there was no difference in viral shedding and possible transmission capability between the groups, although the latter was not assessed. This may be explained because IAV can employ multiple enzymes for HA cleavage and fusion activation in the host (Hatesuer et al., 2013) and in the absence of TMPRSS2, other proteases might compensate. IHC staining revealed that the IA/20 H1N1 strain infected cells throughout the respiratory tract.

The lesions induced by IAV in the respiratory tract reflect not only the damage done by the virus during infection, but also the host’s innate inflammatory response during an acute respiratory tract infection. The inflammatory response is signaled by cytokines and chemokines to initiate development of specific immunity. While the response leads to resolution of the infection, the innate antiviral reaction also mediates inflammation and contributes to clinical signs (i.e., labored breathing, fever, and anorexia) (Hayden et al., 1998; Van Reeth et al., 2002). Many of the cytokines tested were present in significantly higher levels in the lung lavage of WT pigs compared to the *TMPRSS2* ^*−/−*^ pigs. Interferons are upregulated early in antiviral responses in swine. While IFN-α is produced by dendritic cells and IFN-γ by T cells and natural killer (NK) cells upon antigen stimulation, both play many immunoregulatory roles during early stages of influenza infection (Suradhat et al., 2001; Van Reeth et al., 2002; Barbe et al., 2010). Interleukins are another family of cytokines with potent immunomodulatory properties. IL-1β is produced mainly from antigen presenting monocytes and macrophages and strengthens the response by activating T and B cells and increasing additional cytokine production (Tuo et al., 1996). IL-6 has a significant influence on the development of adaptive immunity and impacts influenza disease severity in animals and humans (Lauder et al., 2013). IL-12 drives the development of cell-mediated immunity (Carter and Curiel, 2005). IL-4 also regulates antibody production, inflammation, and activation of T cells (Brown and Hural, 2017). Conversely, IL-10, a repressor of the proinflammatory response, was also present in higher levels in the lung of WT pigs. This cytokine increases through feedback mechanisms in response to inflammation and is essential for tissue-healing (Ouyang et al., 2011). Therefore, the reduced lung lesions in the *TMPRSS2* ^*−/−*^ pigs were likely associated with a dampened pro-inflammatory response. This was consistent with cytokine levels reported in a *TMPRSS2* knockout mouse model (Iwata-Yoshikawa et al., 2019). A small-scale genome-wide association study (GWAS) demonstrated that elevated TMPRSS2 expression in humans resulted in more severe outcomes of pdm09 influenza disease (Cheng et al., 2015). The lack of *TMPRSS2* expression may result in pigs that experience less inflammation and subsequently reduced clinical signs when infected with IAV. As a result, the overall consequences of disease and subsequent production losses may be decreased.

We acknowledge that our study was limited by the low number of *TMPRSS2* ^*−/−*^ pigs available for IAV challenge; however, we demonstrated the need to investigate disease dynamics in the natural host as our results in the swine model differed from the mouse model. This is particularly critical in the case of IAV, as swine are a natural host. Previous studies regarding host genetic determinants of IAV susceptibility and resistance via alterations in genes that control innate immunity were reported in mice and humans. For instance, *Mx* genes and their corresponding products are a group of interferon-inducible antiviral proteins that have been extensively investigated as suppressors of IAV infection (Palm et al., 2010; Haller and Kochs, 2020). *In vitro*, fibroblast cells taken from genetically modified pigs with increased Mx1 expression exhibited reduced IAV proliferation (Yan et al., 2014). Other studies indicated that this inhibition of viral replication was dependent on the Mx1 protein function and not on interferon induction (Nakajima et al., 2007). There are also host genetic determinants that influence IAV host range. One of the major barriers to avian IAV replication in mammalian cells is the incompatibility of the viral polymerase with acidic nuclear phosphoproteins (ANP). ANPs are host cellular proteins utilized by the viral polymerase for replication of the viral genome and propagation of infectious virus in host cells. This was demonstrated by knockdown of ANP in human cells, reducing IAV replication and transcription (Staller et al., 2019). Porcine ANP32A showed greater avian IAV polymerase activity than ANP32B, and host-specific sequences involved in the interaction were identified that can be targeted for gene editing ANP32A in swine (Zhang et al., 2020). Overall, our findings highlight the use of pigs as natural hosts for investigating mechanisms of IAV susceptibility and generating disease-resistant pig models for mitigating influenza risk. Future studies will be aimed at investigating other host cell proteases that may have compensated for the loss of *TMPRSS2* expression in this single knockout model or may result in an additive effect on IAV resistance in pigs. In addition, pathogenesis, and transmission studies of distinct IAV subtypes and strains in *TMPRSS2* ^*−/−*^ model pigs are warranted as we were limited to only one H1N1 strain due to availability of the knockout pigs.

## Data Availability

The original contributions presented in the study are included in the article/Supplementary Material or at the USDA Ag Data Commons at 10.15482/USDA.ADC/1529793, further inquiries can be directed to the corresponding authors.
