# Supplementary material for: Pigs lacking TMPRSS2 displayed fewer lung lesions and reduced inflammatory response when infected with influenza A virus
Source: Front Genome Ed. 2024 May 31;5:1320180. doi: 10.3389/fgeed.2023.1320180 (PMC11176495; doi:10.3389/fgeed.2023.1320180)
Supplement: Supplementary file 1 [file DataSheet1.docx]

Supplementary Material

# Supplementary Tables

**Table S1**. Primers used for genotyping and RT-PCR

| **Primer Name** | **Primer 5'-3'** |
| --- | --- |
| *TMPRSS2* Genotyping For | CTGCAGCGAAGGCTAAG |
| *TMPRSS2* Genotyping Rev | CACCATCCTGCCTCTATCT |
| *TMPRSS2* RT-PCR For | CCAAATACTTACCTGTACCTGATGC |
| *TMPRSS2* RT-PCR Rev | GGGTGGTACTGAGCCGGATA |
| *ACTG* RT-PCR For | GTGGACATCAGGAAGGACCTCTA |
| *ACTG* RT-PCR Rev | ATGATCTTGATCTTCATGGTGCT |

**Table S2.** Daily body temperatures

| Body temperatures ℃ | |  |  |  |  |  |  |  |  |
| --- | --- | --- | --- | --- | --- | --- | --- | --- | --- |
| Pig # | Group | -2 DPI | -1 DPI | 0 DPI | 1 DPI | 2 DPI | 3 DPI | 4 DPI | 5 DPI |
| 876 | Negative Controls | 38.9 | 38.8 | 38.3 | 37.9 | 38.6 | 38 | 38.2 | 38.1 |
| 877 | Negative Controls | 40.1 | 39.7 | 39.7 | 39.4 | 39.5 | 39.4 | 39.5 | 38.6 |
| 878 | Negative Controls | 40 | 39.8 | 39.5 | 39.4 | 40 | 39.3 | 39.4 | 39.5 |
| 879 | Negative Controls | 40.3 | 39.7 | 39.8 | 39.4 | 39.8 | 39.5 | 39.5 | 39.5 |
| 880 | Negative Controls | 40 | 39.3 | 39.5 | 38.9 | 39.1 | 39.1 | 39.2 | 38.8 |
| 926 | Wildtype Controls | 39.3 | 39.2 | 38.6 | 41.1 | 39.8 | 39.8 | 39.7 | 39.2 |
| 927 | Wildtype Controls | 39.4 | 39 | 39.1 | 41.4 | 40.2 | 40.3 | 40.2 | 40 |
| 928 | Wildtype Controls | 39.5 | 39.4 | 39.5 | 42 | 40.1 | 39.5 | 41 | 39.9 |
| 929 | Wildtype Controls | 39.9 | 39.4 | 39.7 | 40 | 39.7 | 39.5 | 39.1 | 39.5 |
| 930 | Wildtype Controls | 40 | 39.5 | 39.8 | 40.8 | 39.8 | 40 | 39.5 | 39.9 |
| 931 | Wildtype Controls | 38.9 | 38.8 | 38.9 | 40.5 | 40.1 | 37.6 | 38.3 | 39 |
| 932 | Wildtype Controls | 38.9 | 39 | 38.6 | 39.4 | 39.7 | 39.5 | 39 | 38.6 |
| 933 | Wildtype Controls | 39.4 | 39.1 | 38.9 | 40.2 | 39.8 | 38.6 | 40 | 39 |
| 934 | Wildtype Controls | 40.1 | 40.7 | 39.7 | 41.2 | 40.4 | 39.3 | 39.4 | 40.1 |
| 935 | Wildtype Controls | 40.1 | 40 | 39.5 | 41.8 | 40.1 | 39 | 41.1 | 40.7 |
| 1531 | *TMPRSS2^-/-^* | 39.4 | 38.9 | 38.9 | 39 | 41.9 | 39.1 | 39.3 | 39.7 |
| 1532 | *TMPRSS2^-/-^* | 40.1 | 40.3 | 40 | 39.4 | 40.4 | 41 | 39.3 | 40.1 |
| 663 | *TMPRSS2^-/-^* | 39.5 | 39.3 | 40 | 39.4 | 40.4 | 39.9 | 39.7 | 39.8 |
| 664 | *TMPRSS2^-/-^* | 39.7 | 39 | 38.9 | 38.9 | 40.4 | 40.2 | 39.4 | 40.2 |
| 665 | *TMPRSS2^-/-^* | 40.4 | 40.8 | 40.7 | 40.1 | 40.2 | 40.2 | 39.9 | 40 |
| 666 | *TMPRSS2^-/-^* | 40.5 | 39.3 | 40.2 | 40.8 | 40.4 | 40.7 | 40.4 | 40.3 |
| 668 | *TMPRSS2^-/-^* | 39.8 | 39.7 | 39.7 | 40 | 39.9 | 40.2 | 40.4 | 39.9 |

# Supplementary Figures

**Fig. S1. Genotyping and reverse transcription PCR of experimental *TMPRRS2* knockout and Wild type (WT) control pigs**. Top panel: PCR of genomic DNA confirms deletion and loss of exon-2 sequence in the *TMPRRS2* knockout pigs compared to the WT pigs as identified by the decrease in the band size. Middle panel: Reverse transcription (RT)-PCR of RNA extracted from lung biopsies from *TMPRRS2* knockout pigs and WT pigs using primer specific to deleted exon 2 and exon 3 confirmed loss of the gene fragment compared to the WT controls. Bottom panel: RT-PCR of RNA extracted from lung biopsies from *TMPRRS2* knockout pigs and WT pigs using primers specific to a house keeping gene gamma-actin confirming integrity of whole RNA and successful cDNA synthesis from all samples.

MALNSGSRPGVGPYYENHGYQPESVYPPQPPGAHRPYGAYPAQYHPPSVPQYAPRVQTHASTPAVVVSRQPKPRSRT

MCSSKTKKALCITFALGAILAGAVLATVLLWKFMEKKRCSTPEMECGSSGTCISPSHWCDGILHCPGGEDENQCVRLYGPNFILQVYSAQRKSWYPVCQDDWTENYGRAACQDMGYRNSFFSSQGIADDSGATSFMKLNKSANNMDLYKKLYHSDVCTSNTVVSLRCIECGVSGKMSNRQSRIVGGSSAALGDWPWQVSLHVQGIHICGGSIITPDWIVTAAHCVEEPLNNPKIWTXFAGILRQSFMFYGSGYRVAKVISHPNYDPKTKNNDIALMKLQTPMTFNDKVKPVCLPNPGMMLEPTQSCWISGWGATYEKGKTSEVLNAAMVRLIEPWSCNSKQVYNNLITPAMICAGYLQGSVDSCQGDSGGPLVTLKSSIWWLIGDTSWGSGCAKAYRPGVYANVTLFTDWIYRQMRANS

Deleted region: MALNS (including start codon)

Region-untranslated

**Fig. S2. Predicted alteration of the open reading frame and altered amino acid and protein sequence in *TMPRRS2* knockout pigs.** Deletion of exon 2 results in the loss of the canonical start codon and 5 amino acids MALNS (including start codon) and loss of translation up to 77 amino acids (highlighted in yellow).


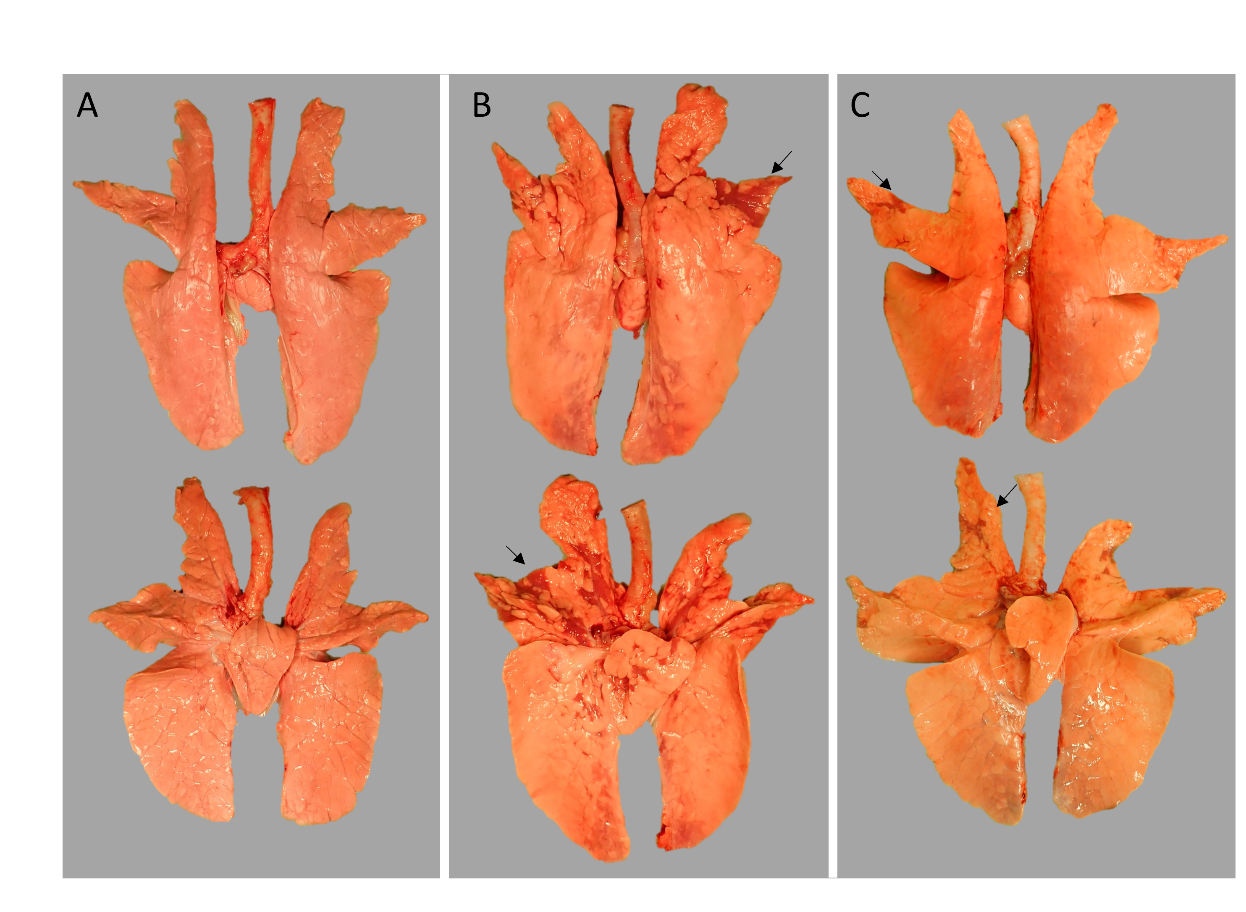


**Fig. S3. Macroscopic lung lesions.** Representative lungs were chosen based on proximity to the group mean score and are arranged in order from negative controls (A) to wildtype (B) and TMPRSS2^-/-^ (C) pigs. Black arrows point to multifocal and coalescing areas of consolidation, consistent with acute Influenza A infection.


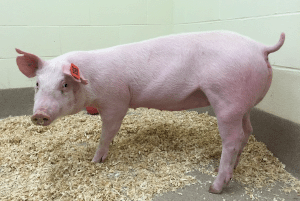


**Fig. S4.** **A pig possessing a knockout of the *TMPRSS2* gene.** Donated by the National Swine Resource and Research Center, University of Missouri. Reference: Whitworth KM, Benne JA, Spate LD, Murphy SL, Samuel MS, Murphy CN, Richt JA, Walters E, Prather RS, Wells KD. 2017. Zygote injection of CRISPR/Cas9 RNA successfully modifies the target gene without delaying blastocyst development or altering the sex ratio in pigs. Transgenic Res. 2017 Feb;26(1):97-107. doi: 10.1007/s11248-016-9989-6. Epub 2016 Oct 15. PMCID: PMC5247313.
